# Crossing the Border: Molecular Control of Motor Axon Exit

**DOI:** 10.3390/ijms12128539

**Published:** 2011-11-29

**Authors:** Arlene Bravo-Ambrosio, Zaven Kaprielian

**Affiliations:** 1 Dominick P. Purpura Department of Neuroscience, Albert Einstein College of Medicine, Bronx, NY 10461, USA; E-Mail: Arlene.Bravo@med.einstein.yu.edu; 2 Department of Pathology, Albert Einstein College of Medicine, Bronx, NY 10461, USA

**Keywords:** motor axon exit, axon pathfinding, spinal cord, motor exit point, dorsally-exiting motor neuron, ventrally-exiting motor neuron, Nkx2.9, Robo, Slit

## Abstract

Living organisms heavily rely on the function of motor circuits for their survival and for adapting to ever-changing environments. Unique among central nervous system (CNS) neurons, motor neurons (MNs) project their axons out of the CNS. Once in the periphery, motor axons navigate along highly stereotyped trajectories, often at considerable distances from their cell bodies, to innervate appropriate muscle targets. A key decision made by pathfinding motor axons is whether to exit the CNS through dorsal or ventral motor exit points (MEPs). In contrast to the major advances made in understanding the mechanisms that regulate the specification of MN subtypes and the innervation of limb muscles, remarkably little is known about how MN axons project out of the CNS. Nevertheless, a limited number of studies, mainly in *Drosophila*, have identified transcription factors, and in some cases candidate downstream effector molecules, that are required for motor axons to exit the spinal cord. Notably, specialized neural crest cell derivatives, referred to as Boundary Cap (BC) cells, pre-figure and demarcate MEPs in vertebrates. Surprisingly, however, BC cells are not required for MN axon exit, but rather restrict MN cell bodies from ectopically migrating along their axons out of the CNS. Here, we describe the small set of studies that have addressed motor axon exit in *Drosophila* and vertebrates, and discuss our fragmentary knowledge of the mechanisms, which guide motor axons out of the CNS.

## 1. Introduction

Mammals rely on motor neurons (MNs) to transmit information from the central nervous system (CNS) to our peripheral organs and somatic muscles, to establish autonomic control of visceral functions and to coordinate musculoskeletal movement, respectively. Unique among all other classes of CNS neurons, axons of developing MNs establish such connections by breaching the basal lamina surrounding the neural tube and emerging from the confines of the CNS. Just like cars traveling on a freeway, motor axons must ultimately exit to reach their final destination. Just as exit signs are positioned on freeways, motor axon exit points are located at well-defined positions along the margin of the CNS. However, breaks in the basal lamina at these positions that would facilitate motor axon exit, analogous to exit ramps for cars, have not been described. Accordingly, how MN axons cross the CNS:PNS interface remains a major unanswered question.

Once in the periphery, MN axons follow a stereotypical trajectory to synapse with muscle targets. Defined by their particular muscle targets, MNs are classified into three categories: (i) somatic MNs, which directly innervate skeletal muscle; (ii) specific classes of visceral MNs, also known as branchiomotor neurons, which directly innervate branchial (pharyngeal) arch-derived muscles; and (iii) generic visceral MNs, which indirectly innervate cardiac/smooth muscles or various glands. Despite the significant progress made in elucidating mechanisms that regulate MN subtype diversification, motor axon guidance to specific muscle targets, and the formation of sensory-motor connections, how motor axons leave the CNS remains a poorly understood phenomenon. In this review, we will focus on one of the most important, but poorly understood aspects of MN development—molecular mechanisms that control the projection of motor axons towards and out of the CNS—but we begin by describing the development of both somatic and branchial MNs, highlighting the cellular and molecular mechanisms that specify these particular classes of MNs.

## 2. Motor Neuron Subtypes: Dorsally- and Ventrally-Exiting Motor Neurons

**Figure 1:**
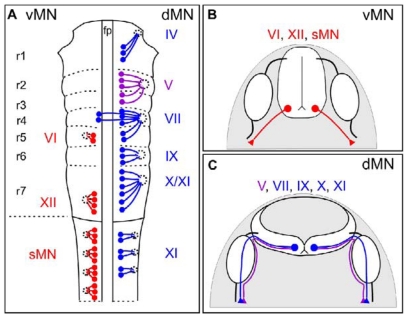
Motor neuron subtypes and the projections of their axons out of the CNS. (**A**) Schematic of motor neuron nuclei in the developing brainstem (rhombomere (r), r1 to r7) and spinal cord. vMNs are indicated in red on the left, whereas dMNs are indicated in blue on the right of the schematic. Trigeminal (V) motor nuclei are shown in purple. Each cranial motor nuclei is numbered in roman numerals, e.g., CN XI. Abbreviations: fp, floor plate; sMN, spinal motor neuron; (**B**) Axonal projections of vMNs in the hindbrain (VI, XII) and spinal cord (sMN) are shown in red; (**C**) Axonal projections of dMNs (VII, IX, X, XI) and trigeminal (V) dMNs are shown in blue and purple, respectively. Note that axons extending from trigeminal dMNs avoid sensory ganglia (white ovals), while axons of other dMN invade these ganglia [6].

Motor neuron subtypes are categorized by the settling positions of their cell bodies and the trajectories that their axons follow to exit the CNS. A particularly clear distinction among MN subtypes is the relative position of their exit points along the margins of the brainstem and spinal cord; ventrally- (vMNs) and dorsally- (dMN) exiting MNs extend through ventral and dorsal points, respectively (Figure 1) [1–8]. Insights from vertebrate evolution may explain why MN axons project through either dorsal or ventral motor exit points. In primitive chordates such as amphioxus, axons of MNs and Rohon Beard sensory neurons project through dorsal exit points within the spinal cord [9]. As vertebrates such as the lamprey emerged, vMNs replaced those that exited at more dorsal positions within the spinal cord. However, axons of cranial MNs located within the vertebrate hindbrain retained their ancestral trajectory and emerged from the CNS at dorsal exit points [9]. Notably, in mouse embryos, the state of Cxcl12-Cxcr4 signaling appears to facilitate the projection of vMN axons to ventral as opposed to dorsal exit points (Table 1) [6]. Although speculative, the evolution of this ligand-receptor pair (Cxcl12-Cxcr4), across vertebrates may provide insights into why and how subtypes of motor axons grow towards precisely positioned exit points.

Both dMN and vMN subtypes are located in the developing brainstem and spinal cord and extend their axons to branchial arch-derived and skeletal muscles, respectively. Another distinguishing feature of vMN and dMN is the location of their cell bodies along the D-V axis of the hindbrain/spinal cord; vMN settle in more ventral positions, whereas dMN migrate dorsally and settle at dorsal locations along the D-V axis of the hindbrain/spinal cord. The majority of dMNs reside within the brainstem and include neurons whose axons form cranial nerves (CN) IV, V, VII, IX, X, XI (cranial component) (Figure 1) [7,8]. Collectively, these particular CN play an important role in controlling head, eye, jaw, and neck movement, feeding, speech, and facial expressions [7,8]. The sole dMN population within the spinal cord is spinal accessory motor neurons (SACMN). SACMN are the only spinal MNs whose axons emerge from a dorsally-positioned motor exit point termed the lateral exit point (LEP) and assemble into the spinal accessory nerve (SAN), which comprises the spinal component of CN XI and innervates a subset of neck and back muscles [2,3,5,21].

In addition to the positioning of their cell bodies and the trajectories that their axons follow to project out of the CNS, vMN and dMN can also be distinguished by the complement of transcription factors (TFs) they express. Branchiomotor neurons, a class of dMN, express the homeodomain TFs, Phox2a and Phox2b, which are required for the proper specification of this particular MN subtype ([17,22] and data not shown). Consistent with its role in dMN differentiation, Phox2b is required for the development of branchiomotor neurons, including SACMN [17,22]. dMN also express the T-box transcription factor, Tbx20, which is required for proper cell body migration of cranial MNs within the vertebrate hindbrain [23]. In contrast, developing vMNs express neither Phox2b nor Tbx20 and, instead, selectively express the homeobox gene *Hb9* ([24] and data not shown). Hb9 has an essential role in maintaining the identity of postmitotic vMN; in mice lacking Hb9, vMNs are generated in normal numbers and on schedule, however, they subsequently acquire a V2 interneuron identity [24].

## 3. Motor Axon Exit Points

As noted above, motor neuron subtypes can be distinguished by the path that their axons take to exit the neural tube [25–27]. During embryonic development, motor exit points are prefigured by neural crest derivatives, referred to as boundary cap (BC) cells [16,18,28,29]. Boundary cap cells not only reside at vMN [16,18,29] and dMN ([28] and data not shown), but also at sites through which sensory axons enter the spinal cord or the dorsal root entry zone [30]. *Krox-20* [31,32], a TF that belongs to the zinc finger family, cadherin-7 (Cad-7), a cell surface adhesion molecule and a member of type II cadherins [33,34], *C-Lingo-1* [35], erythropoietin receptor [36], and monoamine oxidase B [37], are each BC markers in mammals. Given that BC cells are strategically positioned at motor axon exit points, it seemed reasonable to suspect that these cells regulate exit point-related MN guidance events [28]. The role of BC cells at motor exit points was investigated using three different types of manipulations/model systems: (1) surgical ablation of neural crest cells in chick embryos; (2) *Splotch* (*Pax3* mutant) mice embryos whose neural crest cells fail to delaminate from the trunk region of the neural tube [38–40]; (3) the *selective* ablation of Krox-20-expressing BC cells by targeting diphtheria toxin to the *Krox20* locus [29]. Notably, in all three *in vivo* models, the absence of BC cells failed to disrupt the pathfinding of vMN axons but, remarkably, their cell bodies ectopically migrated out of the CNS [29].

**Figure 2:**
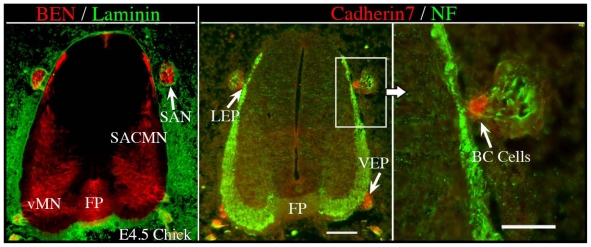
Cadherin 7-expressing BC cells are located at both the lateral and ventral exit points in chick embryos. Cervical spinal cord-containing transverse cryosections derived from E4.5 chick embryos were doubled-labeled with either anti-Ben and anti-Laminin (**Left**) or anti-Cadherin 7 and anti-NF (**Middle**, **Right**), and the appropriate secondary antibodies. (**Left**) BEN is expressed by both SACMN and vMN at this developmental stage, the FP and the SAN, which is positioned outside and adjacent to the spinal cord. Anti-Laminin labeling demarcates the margin of the spinal cord. (**Middle**) Cadherin 7-expressing BC cells are located at both the LEP and VEP. In this panel, the SAN is labeled by anti-NF. (**Right**) A higher magnification view of the boxed area in the middle panel. SACMN, spinal accessory motor neurons; SAN, spinal accessory nerve; vMN, ventral motor neurons; FP, floor plate; LEP, lateral exit point; VEP, ventral exit point; BC cells, boundary cap cells. Scale bar in middle panel, 100μm, applies to the left and middle panels. Scale bar in right panel, 50 μm.

Consistent with previous findings at ventral exit points [29], we have observed that Cadherin-7- and Krox-20-expressing BC cells reside outside of the spinal cord and adjacent to *dorsal* exit points, including the lateral exit point (LEP) in chick and mouse embryos, respectively (Figure 2 and data not shown). To assess the *in vivo* role of BC cells in SACMN development, we analyzed *Krox20^DT^* mouse embryos, by co-labeling transvserse cryosections with anti-BEN, a selective marker for SACMN, and anti-islet1 or anti-phox2a, which are markers for all MNs or branchiomotor neurons (dMN), respectively. This revealed that SACMN axon pathfinding toward, and out of the spinal cord through the LEP was unaltered in these Krox-20-expressing BC cell-lacking animals. Notably, however, a considerable number of BEN-/Islet1- and BEN-/Phox2a-labeled SACMN cell bodies migrated out of the spinal cord and accumulated within the SAN in *Krox20^DT^* embryos (data not shown), and we observed a corresponding decrease in the number of BEN-/Islet1- and BEN-/Phox2a-labeled SACMN within the spinal cord of these animals (data not shown). Taken together, our findings are consistent with the behavior of vMN neurons and their axons in the absence of ventral exit point-associated BC cells [29], indicating that these unique cells confine both vMN and dMN cell bodies to the spinal cord.

It has recently been established that BC cells confine vMN cell bodies to the vertebrate spinal cord through repulsive semaphorin-plexin interactions [16,18,41]. Specifically, selective knockdown of *Neuropilin 2* in vMNs results in aberrant migration of their cell bodies out of the CNS along ventral nerve roots [16]. Consistent with these findings, vMN somata were observed at ectopic positions in the PNS of mice lacking Npn-2. Furthermore, BC cells express Sema6A, a transmembrane semaphorin, and *Sema6A*-deficient mice phenocopy *Npn2* mutants, in that both exhibit an ectopic vMN cell body migration phenotype. Collectively, these interesting observations support a model in which semaphorin-mediated repellent interactions between Neuropilin2-expressing vMNs and Sema6a-positive BC cells constrain vMN cell bodies to the CNS, by opposing the forces exerted by exiting vMN axons on their cell bodies (Table 1). Consistent with Sema6a’s role in regulating vMN somata positioning, an independent study showed that Sema6a-PlexinA1 signaling is required for BC cells to cluster at the PNS/CNS border and, thus, prevents vMN cell bodies from inappropriately translocating out of the CNS (Table 1) [18].

**Figure 3:**
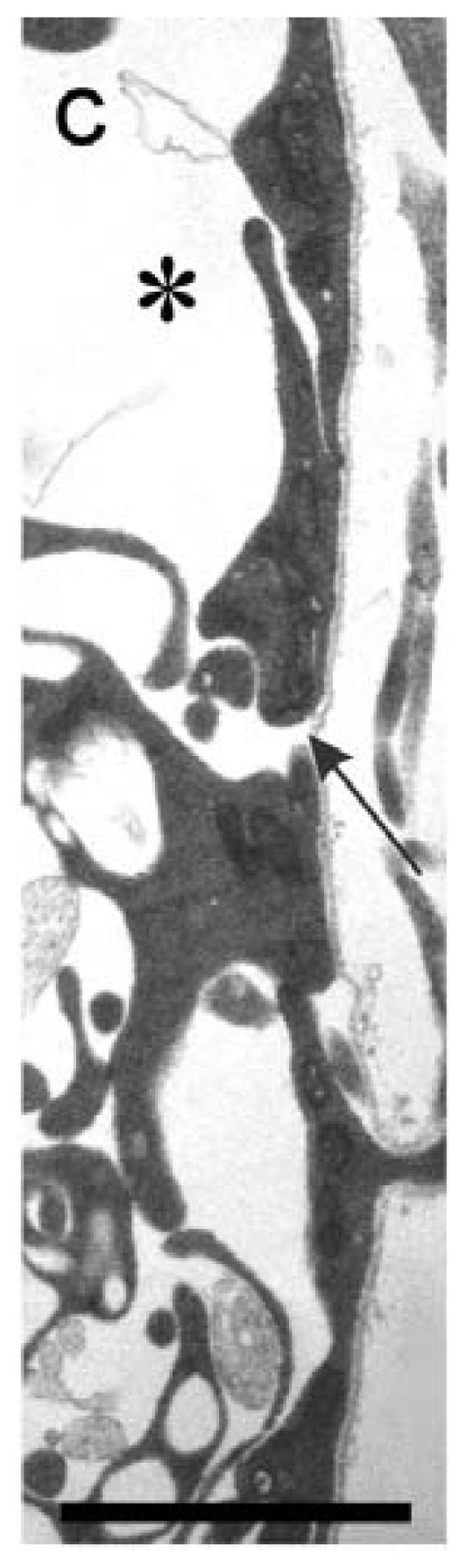
Electron micrograph of a section of neural tube derived from an E11 rat embryo at a presumptive motor exit point. A transverse section of the neural tube shows a thin layer of uninterrupted basal lamina that overlies glial end-feet, which form a conspicuous gap at a presumptive motor exit point. Black arrow, presumptive motor exit point; black asterisk, space found within presumptive white matter. Scale bar: 2 μm [42].

Contrary to the observations that BC cells prefigure motor exit points, ultrastructural studies reveal that cellular features indicative of these cells are, in fact, not present at motor exit points just prior to the developmental stages when motor axons leave the CNS [42]. Specifically, electron microscopy (EM) failed to identify conspicuous clusters of morphologically unique cells at the CNS/PNS border (also referred to as the transition zone; TZ) in the vicinity of presumptive motor exit points within the hindbrain and spinal cord, when motor axons grow out of the vertebrate CNS. Instead, aggregates of these cells were only detected *after* motor axons leave the neuroepithelium and at considerable distances from exit points or the TZ [42,43]. These findings are in sharp contrast to observations made at sensory axon entry sites, where clusters of BC cells clearly prefigure these zones at the time when axons enter the CNS. In addition, EM micrographs revealed that an uninterrupted coat of basal lamina that covers a discontinuous layer of glial end-feet, which forms a thin barrier surrounding the neural tube termed the glial limitans, is associated with exit points, and gaps between glial end-feet at exit points are thought to facilitate the emergence of motor axons from the CNS (Figure 3) [42].

Since “gaps” between glial end-feet are strategically positioned at motor exit points, it seems possible that motor axons may project through these discontinuities to eventually exit the CNS. It is interesting to note in this regard that the complexity of SACMN growth cones is dramatically reduced such that they become slender and elongated as they reach their dorsally located exit point [3]. Such morphological changes in motor axon growth cones as they project out of the CNS would allow them to grow through small “gaps” formed by glial end-feet at motor exit points (see above).

### 3.1. Motor Exit Point Chemoattractants

A long held view has been that motor exit point-derived chemoattractants guide motor axons towards the positions at which they emerge from the CNS [29,44–47]. There are several lines of evidence supporting the existence of target (e.g., dermomyotome, limb bud, cranial sensory ganglia, branchial arches) derived chemoattractants for ventrally- and dorsally-exiting motor axons, including fibroblast growth factor (FGF) [48], hepatocyte growth factor (HGF) [46,49], and possibly ciliary neurotrophic factor (CNTF), brain-derived neurotrophic factor (BDNF), and cardiotrophin 1 (see [50]. The role of FGF as a dermomyotome-derived chemoattractant for a subclass of vMNs (MMCm) in vertebrates has been previously described [48]. A number of studies provide evidence that limb-derived mesenchymal tissue are capable of stimulating neurite outgrowth from spinal cord explants *in vitro* [51,52]. Although the identities of these chemoattractive forces are largely unknown, the vertebrate limb bud expresses candidate neurotrophic factors that support the survival of developing MNs and that may also stimulate the outgrowth of their axons. For example, limb bud derived-HGF attracts motor axons *in vitro*, however, motor axons are still capable of projecting to their target cells in *HGF*-deficient mice, raising the possibility that other (neurotrophic) chemoattractive factors co-exist and operate in a redundant manner within the developing limb. Consistent with this finding, antibodies to neurotrophins (NGF, BDNF, and NT-3) co-cultured with limb slices derived from mouse embryos revealed that these factors are required for the extension of motor axons to their associated limb targets [53]. Whether or not the target-derived chemoattractants described above are expressed at motor exit points remains to be determined and exit point-derived chemoattractants have yet to be identified for any subtype of MN. Nevertheless, studies in the embryonic chick hindbrain demonstrated that motor axons re-direct their growth and appropriately project towards their exit points after reversing the rostrocaudal polarity of particular rhombomeres [54]. In addition, both exit point-derived isthmic tissue, which normally expresses *FGF8* mRNA, and FGF8 protein, is capable of attracting trochlear motor axons *in vitro* [55].

### 3.2. Positioning of Motor Exit Points

Motor exit points are segmentally reiterated along the rostrocaudal neural axis during embryonic development; however, our understanding of the mechanisms responsible for establishing their positions is fragmentary at best. Studies carried out in zebrafish embryos indicate that the *sidetracked* (*set*) gene, which was isolated from a genetic screen for molecules regulating motor axon pathfinding [56], has a role in positioning motor exit points (Table 1). In wild type zebrafish embryos, axons from three primary MNs: the caudal primary (CaP), middle primary (MiP), and rostral primary (RoP), exit the spinal cord through a single exit point formed beneath the CaP cell body and located at the midpoint of the overlying somite. It is well-documented that CaP growth cones are the first to exit the spinal cord, pioneering a common path that is subsequently followed by MiP and RoP growth cones/axons [12]. Once all motor growth cones reach the distal end of this common path, also referred to as a choice point, they project along divergent paths to their target myotome regions. In contrast, *sidetracked* zebrafish mutants display dramatic motor axon exit defects; both MiP and RoP appropriately project in a caudal direction but bypass or extend rostrally away from their exit point, or even exit at ectopic positions directly beneath their cell bodies. Subsequently, it was shown that the gene disrupted in *sidetracked* mutants encodes the Semaphorin receptor, Plexin-A3 [19]. Using single-cell labeling and time-lapse microscopy, it was also shown that Plexin-A3 acts cell autonomously to direct MiP and RoP motor growth cones to their exit point, suggesting that posterior somite-derived semaphorins trigger repulsive Plexin-A3 signaling, which regulates MiP/RoP motor axon exit.

Additional findings from zebrafish embryos suggest that the positions of primary MN cell bodies prefigure motor exit points. As mentioned above, axons of primary neurons (CaP, MiP, and RoP) exit the spinal cord immediately beneath the CaP cell body. Semaphorin-Neuropilin signaling plays an important role in establishing the proper positioning of CaP cell bodies and, consequently, motor exit points [15]; the position of CaP, which expresses Neuropilin1a, was disrupted by antisense knockdown of *Neuropilin1a* and this gave rise to ectopic motor exit points. In complementary studies, knockdown of somite-derived *Sema3ab*, a class III semaphorin ligand for Neuropilin1a, resulted in an irregular distribution of CaP cell bodies and an increased number of ectopic motor axon exit points. Taken together, interactions between Neuropilin1a-expressing CaP MNs and somite derived-Sema3ab appear to be required for fine-tuning the position of CaP cell bodies.

CNS-derived glia, which ultimately form the perineurium that ensheaths and protects motor nerves, also prefigure motor axon exit points in Zebrafish embryos [57]. Specifically, in the absence of perineurial cells, which arise from ventral spinal cord-derived glia, MN axons exit the CNS at ectopic positions [57]. MN cell bodies were also observed to inappropriately migrate out of the spinal cord in the absence of CNS-derived perineural glia. Notably, mouse and chick embryos that lack neural crest-derived BC cells exhibit a similar phenotype (see above). Interestingly, in a separate study, it was shown that Zebrafish lacking neural crest cells do not display motor axon exit or MN cell body migration defects, like those observed in embryos lacking CNS-derived glia [58]. These particular observations suggest that, in zebrafish, neural crest cells are not required to confine MN somata to the CNS or for the appropriate projection of motor axons out of the spinal cord. Rather, perineurial cells appear to function as a barrier and regulate motor axon guidance at exit points in these vertebrates. In addition, Kucenas *et al*., 2009 observed that distinct interactions between progenitors of oligodendrocytes and Schwann cells, the myelinating cells of the CNS and PNS, respectively, prevent oligodendrocytes from migrating out of the spinal cord. Specifically, in the absence of peripheral Schwann cells, CNS-derived oligodendrocyte progenitors cross the CNS-PNS interface via ventral nerve roots and myelinate motor axons in the periphery. Taken together, these studies suggest that motor exit points are not only permeable to motor axons, but also to perineural glia cells, and that distinct mechanisms regulate the migration of MN/oligodendrocyte progenitor cell bodies and the guidance of motor axons at exit points.

### 3.3. Signaling Molecules That Control the Projection of Motor Axons to Their Appropriate Exit Points

The FP derived chemorepellents, Slits and Netrin-1, guide cranial motor axons along a dorsally-directed trajectory away from the ventral midline and toward their dorsal exit points, however, the downstream signaling mechanisms remain largely unknown [5,21,59–61]. Recent studies utilized a combination of *in vitro* and *in vivo* approaches to identify molecular components of Slit and Netrin-1 signaling pathways in the context of motor axon pathfinding. Specifically, it was shown that Slit and Netrin-mediated growth cone repulsion depends on retrograde actin flow [13], a process through which actin filaments are constantly being transported from the leading edge by myosin motors including myosin II (reviewed by [62]). Furthermore, Slit and Netrin-1 signaling is mediated by RhoA-kinase (ROCK) and myosin light chain kinase (MLCK), which positively regulate myosin II function by phosphorylating myosin II regulatory light chain (MRLC) [13]. Inhibition of ROCK, MLCK or myosin II in motor explant cultures produces a number of cranial motor axon pathfinding errors, including the inability of these axons to project away from the FP and their aberrant projection into the ventral midline. In complementary studies, genetic manipulation of RhoA, a GTPase protein that activates ROCK or myosin II in chick embryos results in cranial motor axon pathfinding errors, consistent with a loss of FP-mediated repulsion. In addition, de-regulation of RhoA and myosin II function *in vivo* results in branchiomotor axons mis-projecting away from or towards appropriate or incorrect motor exit points, respectively (Table 1). Taken together, these particular findings suggest that RhoA and myosin II are required for dMN axons to navigate towards their appropriate dorsal motor exit points.

## 4. Motor Axon Exit

### 4.1. Projecting into the Periphery

**Figure 4:**
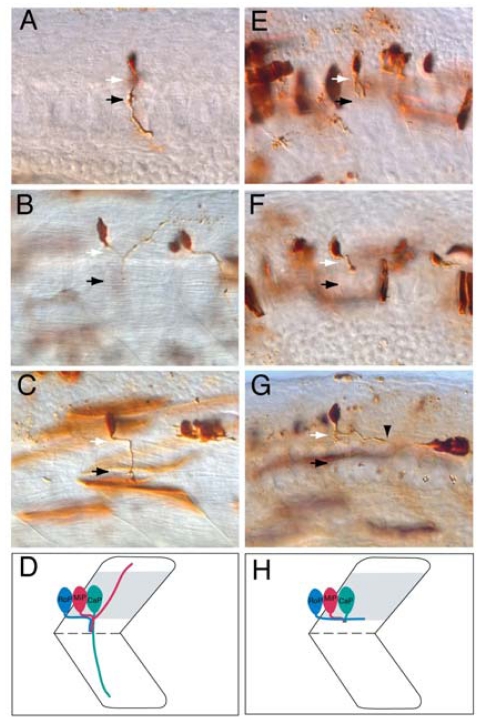
*diwanka* is required for motor axon exit in zebrafish embryos. (**A**–**D**): Wild-type axonal projections of CaP (**A**), MiP (**B**), and RoP (**C**) primary MNs labeled with fixable dyes as previously described [12]. White arrows indicate the lower half of the spinal cord, which is out of the focal plane, whereas black arrows indicate the choice point, the distal end of a common path followed by CaP, MiP, and RoP motor growth cones prior to their divergence into ventral, dorsal and medial myotomal regions, respectively (**D**); (**E**–**H**): In *diwanka* mutants, CaP (**E**) and MiP (**F**) axons extend wild-type projections within the spinal cord, however they exhibit abnormal projections along the common path (black arrow). Notably, the majority of RoP axons fail to exit the spinal cord in *diwanka* mutants and instead extend their axons caudally within the CNS (black arrowhead points to a RoP growth cone) (**G**,**H**) [12].

The initial pathfinding of motor axon growth cones from the CNS to the periphery requires extrinsic guidance cues, yet their identities are only beginning to be identified. The zebrafish *diwanka* gene appears to control this early stage of motor axon pathfinding [12,63]. In diwanka mutants, primary MN growth cones fail to project along a stereotypical route from the spinal cord to somites, raising the possibility that myotome-derived cues delineate this path (Figure 4 and Table 1). Notably, 40% of primary MN axons fail to exit the spinal cord in *diwanka* mutants, suggesting that environmental cues in the periphery promote motor axon exit for a subset of primary MNs. The *diwanka* gene encodes LH3, a myotomally-derived glycosyltransferase, that acts through myotomal type XVIII collagen, a ligand for neural-receptor protein tyrosine phosphatases, which are known to control motor axon pathfinding [11]. These studies propose that LH3 (*diwanka*) catalyzes the addition of a sugar residue to a substrate protein, in this case, type XVIII collagen expressed by myotomal cells. Presumably, these *diwanka*-modified proteins are subsequently secreted from the myotome and deposited along the common motor pathway where they are capable of signaling motor axon growth cones to exit the spinal cord and pioneer into the periphery.

### 4.2. Transcriptional Control of Motor Axon Exit

**Figure 5:**
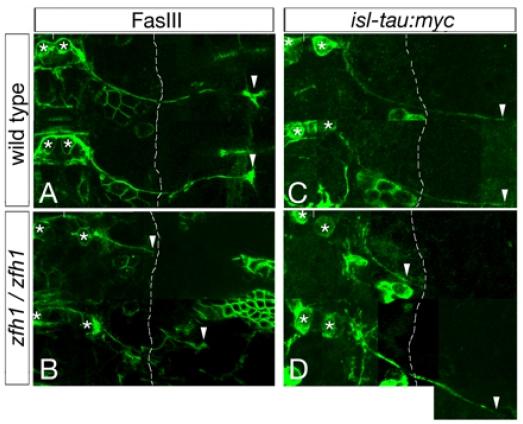
*Zfh1* is required for a subset of motor axons to emerge from the spinal cord. (**A**,**B**) Flat-mounted zebrafish embryos were labeled with either anti-FASIII (**A**,**B**) or anti-myc (**C**,**D**) to visualize the axonal projections of an early-developing intersegmental nerve (ISN) called ISNb. Note, ISNb axons selectively express the *isl-tau:myc* transgene and montages were created by combining images taken from multiple focal planes to visualize the extent of the ISNb nerve. In stage 16 (**A**) and 15 (**C**) wild type embryos, *zfh1-*expressing RP MNs (white asterisk) form an ISNb nerve that extends out and beyond the lateral edge of the CNS (dashed white line). White arrowheads correspond to the terminal end of an ISNb axon. In contrast, a subset of RP MN axons fails to exit the CNS in *zfh1* mutants (**B**,**D**, top), while others extend appropriately to their peripheral muscle field (**B**,**D**, bottom) [20].

**Figure 6:**
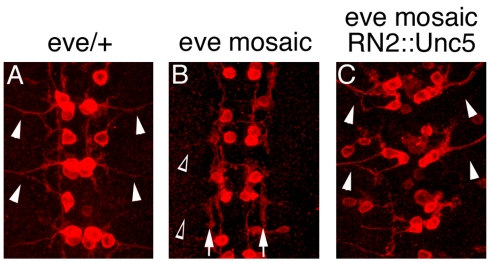
Eve is required for motor axon exit in *Drosophila* and expression of Unc-5 in *eve* mosaic mutants restores motor axon exit defects. (**A**–**C**) Stage-16 *RN2Gal4::CD8GFP* embryos of various genotypes, including *eve*/+ (**A**), mosaic *eve* mutant (**B**), and *Unc-5*-expressing mosaic *eve* mutant siblings (*RN2Gal4, UAS-HAUnc-5*) (**C**) were labeled with anti-GFP to visualize axons of somatic MNs, aCC and RP2. In *eve/+* embryos, aCC and RP2 MNs extend wild type axonal projections to peripheral muscle fields (white arrowheads) (**A**). In contrast, the majority of aCC and RP2 motor axons fail to leave the CNS (89%, *n* = 80 hemisegments) and instead, inappropriately extend their axons longitudinally within the CNS (white arrows) (**B**). However, a small subset of motor axons does manage to exit the CNS (open white arrowhead). Notably, expression of *Unc-5* in *eve* mosaic mutants partially rescues the aberrant motor axon exit phenotype (60% hemisegments, *n* = 100, white arrowheads) (**C**). In all panels, anterior is at the top of the micrograph [10].

A variety of TFs have key roles in regulating the pathfinding behavior of ventrally- and dorsally-exiting motor axons, including their exit from the CNS [64]. For example, in *Drosophila* embryos Nkx6 [14] and Zfh1 [20] are required for vMNs to leave the CNS, whereas, Eve controls the exit of dorsally projecting motor axons (Figures 5, 6 and Table 1) [10]. Supporting a model in which these TFs control motor axon guidance by altering the expression of cell surface effector molecules, Eve and Nkx6 likely regulate the levels of UNC5 [10] and FASIII [14], respectively. In mice lacking both Lhx3 and Lhx4, vMNs adopt a SACMN-like fate and project their axons out of the LEP and, conversely, forced expression of Lhx3 in dMNs re-directs their axons out of the spinal cord through the ventral exit points (Table 1) [4]. Phox2b is also required for dorsal MN development in mouse embryos [22], and, in chick embryos, the mis-expression of Phox2a and Phox2b in interneurons re-specifies these neurons to adopt dMN-like fates and forces their axons to ectopically exit the spinal cord through dorsal exit points (Table 1) [17]. We have previously shown that, in mice lacking Nkx2.9; a homeodomain-containing TF that is expressed in the ventral-most neural progenitor domain of the neural tube where it presumably regulates cell fate decisions [65], SACMN axons appropriately reach the LEP, but fail to leave the spinal cord (Table 1) [5]. Taken together, Lhx3/4, Phox2a/b and Nkx2.9 regulate vertebrate MN/axon development, at least in part, by controlling the expression of particular cell surface proteins/guidance receptors. However, candidate downstream effectors/targets for these MN-associated TFs have yet to be identified.

### 4.3. Spinal Accessory Motor Neurons (SACMN) as a Model System for Elucidating Mechanisms that Regulate Motor Exit Point-Related Guidance Events

**Figure 7:**
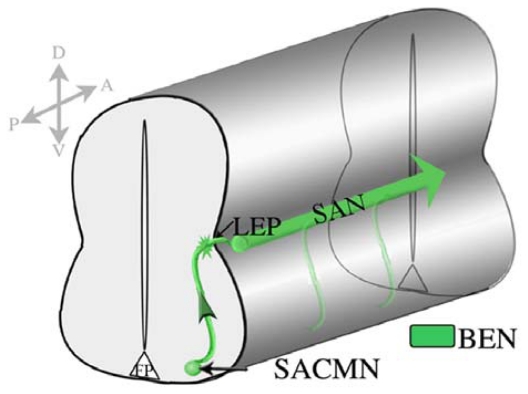
SACMN extend dorsally directed axons towards and through the LEP. Schematic illustrating the axonal trajectory of SACMN and the SAN in the developing vertebrate spinal cord. BEN is selectively expressed on SACMN cell bodies and their axons, as well as on the SAN. D, dorsal, V, ventral, A, anterior, P, posterior, LEP-Lateral Exit Point; SAN-Spinal Accessory Nerve.

In contrast to most previous studies that have focused on the pathfinding of ventrally-exiting spinal MNs, we have utilized dorsally-exiting SACMN as a model system for investigating the mechanisms which regulate motor exit-point related axon guidance events. SACMN cell bodies are found at cervical levels of vertebrate embryos and their axons pathfind along a dorsally-directed trajectory away from the ventral midline of the spinal cord to a position located adjacent to the LEP, midway along the D-V axis [5]. Ultimately, SACMN axons exit through a discrete LEP, turn rostrally and assemble into the SAN, which innervates the sternocleidomastoid and trapezius muscles in the neck and back [2–6]. SACMN represent an ideal model system for elucidating the mechanisms that control exit point-related guidance events in vertebrate embryos since: (1) SACMN are a molecularly homogenous population of dMNs; (2) we have previously identified BEN, an immunoglobulin domain-containing cell surface protein as a selective marker of SACMN cell bodies and their axons in rodent embryos [2,5]; (3) SACMN axons are the sole MN axons that project out of the spinal cord through a discrete LEP; and (4) SACMN axons assemble into a readily identifiable longitudinally projecting SAN outside of the CNS (Figure 7). Moreover, and as mentioned above, we have previously shown that the homeodomain TF, Nkx2.9, is likely required for SACMN axons to project out of the spinal cord [5]. This particular observation prompted us to search for novel downstream effectors of Nkx2.9 that we reasoned might directly facilitate MN axon exit from the mammalian spinal cord.

### 4.4. The Homeodomain Transcription Factor, Nkx2.9, Controls Motor Axon Exit from the Mouse Spinal Cord via Robo-Slit Signaling

We performed candidate-based and microarray screens to identify novel downstream effector molecules of Nkx2.9 that regulate SACMN axon exit from the CNS. Our findings suggest that Nkx2.9 selectively controls the exit of SACMN axons from the spinal cord via the regulation of the Robo2 guidance receptor (unpublished observations). Consistent with such a model, our studies indicate that SACMN axons express Robo2, Slits are present at the LEP, and that SACMN axons fail to exit the spinal cord in mice lacking Nkx2.9, Robo2 and/or Slits (Table 1). We have also shown that Robo2-Slit interactions are required for the proper assembly of SACMN axons into the longitudinally projecting SAN, and that SACMN axons do not stall at the LEP, but rather inappropriately form a longitudinal-projecting ectopic nerve *within* the spinal cord. Whereas previous studies established that Nkx2.9 progenitors are capable of giving rise to interneurons and floor plate cells [65,66], we have shown that they can also generate SACMN within the developing spinal cord, supporting the view that Nkx2.9 acts in a cell autonomous manner to regulate SACMN axon pathfinding.

### 4.5. Homeobox Genes and Motor Axon Exit

Our microarray screen also identified a number of *Hox* genes that are dysregulated in the absence of Nkx2.9. These findings raise the possibility that multiple homeodomain TFs operate in concert to control SACMN axon exit from the spinal cord, perhaps by regulating the expression of Robo2 (see above). Consistent with this hypothesis, Hoxa2 is capable of regulating *Robo2* expression in the vertebrate CNS [67]. In support of the possibility that Hox proteins cooperate with Nkx2.9 to regulate SACMN axon exit, we found that among the five *Hox* genes (*Hoxc8*, *Hoxc6*, *Hoxd8*, *Hoxb9*, *Hoxa6*) retrieved from the microarray screen, *Hoxb9* expression was selectively reduced in *Nkx2.9* null mice (data not shown). Specifically, *Hoxb9* expression in cells located directly above and adjacent to the FP, a domain normally occupied by Nkx2.9-expressing progenitors is reduced, whereas the levels of *HoxC6* appears unaltered (data not shown). These particular findings indicate that Hoxb9 may be a novel putative downstream effector of Nkx2.9. Interestingly, mice with a targeted deletion of the Hoxb1-Hoxb9 locus (HoxbΔ1) exhibit abnormal fusion of cranial nerves IX and X, a phenotype also observed in *Nkx2.9* mutant mice ([65,68] and data not shown). Although these mice do not selectively lack *Hoxb9*, such findings raise the possibility that *Hoxb* genes are involved in cranial motor nerve development. It is also tempting to speculate that Hoxb9 may be a component of an Nkx2.9-dependent transcriptional program that regulates the projection of SACMN axons out of the CNS.

## 5. Perspectives

Although motor axon exit is a key phase of MN development, our understanding of this phenomenon, and especially the underlying molecular mechanisms that operate in vertebrate systems, is fragmentary at best. Accordingly, there is much to be done with respect to identifying the repertoire of molecules that facilitate the projection of motor axons across the CNS:PNS border. Below, we discuss several of the unresolved issues associated with this indispensable phase of MN development that must be addressed in future studies, with an emphasis on how motor growth cones might breach the basal lamina surrounding the vertebrate CNS.

### 5.1. A Novel Role for Robo-Slit Signaling in Motor Axon Exit

Although Robo-Slit interactions are well known to regulate a variety of axon guidance events, our findings described above are the first to implicate Robo-Slit signaling in *motor axon exit* for any model organism [69]. Further, we suggest that attractive or repulsive Robo2-Slit interactions at the LEP may facilitate SACMN axon exit from the spinal cord. Co-culturing SACMN-containing spinal cord explants with LEP-associated tissue would help to differentiate between these two possibilities. Additionally, by co-culturing similar motor explants with a Slit-expressing source it should be possible to determine which Slit(s) evoke a response from Robo2-expressing SACMN axons. It is conceivable that Robo2 facilitates the growth of SACMN axons across a source of LEP-associated Slit via an attractive mechanism. Consistent with this possibility, a positive role for Robo2 in promoting the growth of commissural axons across the Slit-rich midline in the *Drosophila* ventral nerve cord has recently been reported [70–72]. These particular studies hypothesize that Robo2 acts in an attractive manner to promote midline crossing through a source of Slit, potentially by antagonizing Robo1 repulsion [70–72]. Although fly Robo2 is not the orthologue of vertebrate Robo2, this does not preclude a role for vertebrate Robo2 in promoting SACMN crossing at the LEP in an attractive manner [73]. In this regard, and by analogy with the role of Robo3 in facilitating midline crossing in the mouse spinal cord, Robo2 might promote SACMN axon exit by repressing the actions of Robo1 [73,74]. Whereas SACMN do appear to express Robo1, we failed to observe rescue of SACMN axon exit in mice lacking both Robo1 and Robo2 (data not shown). Accordingly, it seems unlikely that Robo2 antagonizes Robo1 signaling in the context of SACMN axon exit.

Although our studies have focused on a specific population of spinal MNs, they may provide insights into a more general understanding of how sensory and motor axons enter and exit the CNS, respectively. For example, our observations might also be relevant to the molecular logic that controls the exit of other classes of motor axons, distributed all along the A-P axis of the developing CNS, including those residing in the hindbrain. In this regard, it is interesting to note that cranial nerves IX and X appear fused in *Nkx2.9* null mice ([65] and data not shown).

### 5.2. Is There a “Molecular Network” That Controls Motor Axon Exit?

Given that a number of molecules are likely to regulate motor axon exit-related events in vertebrates, it seems reasonable to suspect that at least a subset of these molecules operate in concert within a specialized network to promote the exit of motor axons from the developing mammalian CNS. Notbaly, both Nkx2.9 and myotome-derived LH3 are required for MN axons to exit the spinal cord in vertebrates (data not shown and [11,12]). Accordingly, *LH3* expression may be altered in the absence of Nkx2.9, making LH3 a putative downstream effector of Nkx2.9 that directly facilitates SACMN axon exit. Consistent with this hypothesis, *Nkx2.9* mRNA is expressed by the mesoderm, which is the germ layer that ultimately gives rise to myotome, at a very young age (~E7.0) (see [75], Figure 3(D)). Given that Nkx2.9 and Eve regulate dMN axon exit in vertebrates and *Drosophila Melanogaster*, respectively, it is also possible that vertebrate homologs of *Eve, Evx-1* and/or *Evx-2* regulate dMN motor axon exit in mammals. As also noted above, *Hox* genes were retrieved from our microarray screen, making them good candidates for participating in an Nkx2.9-regulated cascade that promotes SACMN axon exit. Interestingly, vertebrate *Hox* genes map closely to *Evx-1* and *Evx-2*, raising the possibility that Nkx2.9 controls SACMN axon exit by regulating the expression of these particular transcription factors “in cis” [76–78].

### 5.3. Crossing the Basal Lamina Border

To ultimately emerge from the CNS, motor axons must somehow break through the basal lamina surrounding the neural tube. Although the studies that we have described in this review, including our own, do not directly address the molecular mechanisms underlying this phase of motor axon pathfinding, it is conceivable that cytoskeletal changes in motor axon growth cones facilitate the projection of motor axons through circumscribed exit points (see above). In addition, the release of proteolytic enzymes, such as particular types of proteases, may promote motor axon exit by generating precisely-positioned breaks in the basal lamina. Consistent with this hypothesis, two families of metalloproteases, the A Disintegrin and Metalloproteases (ADAMs) and matrix metalloproteases (MMPs), have been implicated in regulating axon pathfinding in the developing CNS [79]. Notably, a member of the ADAM family, ADAMTS3, was retrieved from our microarray screen and the corresponding mRNA levels appear to be reduced in the absence of Nkx2.9 (data not shown). It is conceivable that SACMN axons express ADAMTS3 to facilitate their exit from the CNS, however, further studies will be required to address this and other possibilities. Taken together, it seems plausible that Robo-Slit signaling at the LEP normally triggers the expression of ADAMTS3 which, in turn, facilitates the growth of SACMN axons across the CNS:PNS border.

### 5.4. Do Motor Neuron Growth Cones Use Invadopodia to Cross the CNS:PNS Barrier?

The demonstrated ability of tumor cells to degrade extracellular matrix prompted us to ask whether motor axon growth cones can also function in this manner and, if so, do they utilize the same machinery deployed by invasive cancer cells? It is well-established that cancer cells invade layers of extracellular matrix, a key step in tumor metastasis, by extending small localized protrusions, termed invadopodia, which preferentially degrade matrix [80]. Studies addressing the molecular mechanisms underlying the invasive properties of these cancer cells often employ the so-called “invadopodium degradation assay.” This involves plating cells on a culture surface coated with a thin layer of fluorescently-labeled matrix and using fluorescence microscopy to identify regions devoid of fluorescence, which presumably correspond to areas of matrix degraded by the cell [81–84]. As a first step toward determining whether or not MNs are capable of degrading extracellular matrix, we adapted this assay and performed pilot studies by plating neurons derived from the embryonic spinal cord on fluorescent matrix-coated dishes. Our preliminary findings revealed that developing spinal neurons express the invadopodia marker, cortactin, and that these particular cells have the capacity to breakdown extracellular matrix (data not shown). To determine if *motor neurons*, in particular, are capable of degrading matrix, BEN-, Phox2b- and HB9-expressing MNs can be isolated from the spinal cord of BenGFP, Phox2b-eGFP, and Hb9-GFP mice, respectively, via fluorescence activated cell sorting (FACS) [85–88] and tested in the invadopodia assay. Positive results in these experiments would suggest that tumor cells and embryonic spinal MNs employ similar mechanisms to accomplish a fundamentally similar task, namely the degradation of extracellular matrix, for very different purposes.

## 6. Experimental Section

### 6.1. Animals

Expression studies were performed using E4.5 chick embryos purchased from Charles River Laboratories, Avian Products and Services (SPAFAS).

### 6.2. Immunohistochemistry

*Cryosection preparation and immunolabeling*. Whole embryos were fixed in 4% paraformaladehyde (PFA) for 12 h at 4 °C, followed by cryoprotection in 30% sucrose in 4 °C until embryos sank to the bottom of the tube. Embryos were then embedded in optimal cutting temperature compound (Tissue Tek; Sakura Finetek) and stored in the −20 °C. Cryosections (16 μm) were generated using a Leica cryostat (model #; CM3050 S; Leica Microsystems) and collected on Superfrost Plus microscope slides (Fisher Scientific, Cat. No. 12-550-15). Transverse cryosections were rinsed in PBS, post-fixed for 10 min, washed with PBS 1× 5 min, and incubated for 1 h in blocking solution. Sections were subsequently incubated with primary antibody diluted in blocking solution O/N in 4 °C. Following primary antibody incubation, sections were washed 3× 10 min in blocking solution, incubated with the appropriate secondary antibodies, for 1 h, and washed 3× 10 min with blocking solution. The following antibodies were used in this study: anti-BEN/SC1 (DSHB), anti-Cadherin 7 (DSHB), and anti-NF (Sigma).

### 6.3. Photodocumentation

Primary antibody binding was visualized using conventional epifluorescence microscopy (Nikon Eclipse TE300) and images were captured with a digital camera (Optronics) and compatible Magnafire software [5]. In all cases, composites were assembled and annotated using Adobe Photoshop CS3.

## Figures and Tables

**Table 1:** Molecules localized to motor exit points and/or required for motor axon exit.

Name	Organism	Localization	Role in motor neuron development
Cxcl12	Mouse	mesenchyme	Required for a subset of vMN axons to exit through ventral exit points[6]
Cxcr4	Mouse	vMN	Required for a subset of vMN axons to exit through ventral exit points[6]
Eve (TF)	*Drosophila*	dMNs	Required for dMN axons to exit the CNS[10]
LH3 *(diwanka*)	Zebrafish	myotome	Promotes the exit of a subset of MN axons from the CNS[11,12]
Lhx3/Lhx4 (TF)	Mouse	vMNs	Required for vMN specification; sufficient to re-direct dMN axons to and through ventral exit points[4]
Myosin II	Chick	growth cone	Required for a subset of dMN axons to project towards their appropriate motor exit point[13]
Nkx6 (TF)	*Drosophila*	vMNs	Required for vMN axons to exit the CNS[14]
Nkx2.9 (TF)	Mouse	SACMN derived from Nkx2.9 progenitors	Required for SACMN axon exit *
Npn1a	Zebrafish	CaP MN	Required for the proper positioning of motor exit points[15]
Npn2	Chick/Mouse	vMN cell bodies	Required for the confinement of vMN somata to the CNS [16]
Phox2a/Phox2b (TF)	Chick	dMNs	Sufficient to re-specify interneurons into MNs[17]
PlexinA1	Chick	vMN axons	Prevents vMN somata from inappropriately migrating out of the CNS[18]
PlexinA2	Chick	vMN cell bodies	Prevents vMN somata from inappropriately migrating out of the CNS[16]
PlexinA3 (*sidetracked*)	Zebrafish	MiP and RoP MNs	Required for the proper positioning of motor exit points[19]
RhoA	Chick	growth cone	Required for a subset of dMN axons to project towards their appropriate motor exit point[13]
Robo2	Mouse	SACMN axons	Required for SACMN axon exit *
Sema3ab	Zebrafish	Somite	Required for the proper positioning of motor exit points[15]
Sema6A	Chick/Mouse	BC Cells	Prevents vMN somata from inappropriately migrating out of the CNS[16,18]
Slit1/Slit2	Mouse	Lateral exit point-associated cells	Required for SACMN axon exit*
Zfh1 (TF)	*Drosophila*	vMNs	Required for a subset of vMN axons to exit the CNS[20]

The role (or proposed role) of these motor exit point- and motor axon exit-associated molecules is discussed in the text. Abbreviations: CaP, caudal primary MN; dMN, dorsally-exiting motor neuron; MiP, middle primary MN; RoP, rostral primary MN; SACMN, Spinal Accessory Motor Neuron; TF, transcription factor; vMN, ventrally-exiting motor neuron.

* Unpublished observations.
